# Effectiveness of Virtual Reality for Managing Pain, Fear, and Anxiety in Children and Adolescents Undergoing Needle-Related Procedures: Systematic Review with Meta-Analysis

**DOI:** 10.3390/nursrep14030182

**Published:** 2024-09-19

**Authors:** Rocío Cáceres-Matos, Mario Castillo-García, Eleonora Magni, Manuel Pabón-Carrasco

**Affiliations:** 1 Faculty of Nursing, Physiotherapy and Podiatry, University of Seville, 41009 Seville, Spain; rcaceres3@us.es (R.C.-M.); marcasgar29@alum.us.es (M.C.-G.); mpabon2@us.es (M.P.-C.); 2 CTS-1050 “Complex Care, Chronicity and Health Outcomes” Research Group, Universidad de Sevilla, 41009 Seville, Spain; 3 Institute of Biomedicine of Seville (IBiS), 41013 Seville, Spain; 4 CTS-969 “Care Innovation and Health Determinants” Research Group, Universidad de Sevilla, 41009 Seville, Spain

**Keywords:** virtual reality, pain management, fear, anxiety, invasive procedure, adolescent, child, nursing

## Abstract

The most frequently performed invasive procedures in hospitals and healthcare centers are needle-related procedures, such as intravenous cannulation and phlebotomy, and they are identified as the major sources of pain, fear, and anxiety in children and adolescents. The objective of this systematic review was to evaluate the effectiveness of VR as a distraction measure to reduce pain, fear, and anxiety in children and adolescents undergoing needle-related invasive procedures. For this purpose, the CINAHL, Scopus, WOS, and Cochrane Library scientific databases were used. The protocol review was registered in PROSPERO (ID:42024563245), and inclusion and exclusion criteria were applied. Twenty-one studies were included in the systematic review, involving a total of 2663 participants. Significant differences favored the use of virtual reality for the control of pain intensity (WBFSpatients *p* = 0.001; MD = −1.83; 95% CI −2.93 to −0.72; WBFSparents *p* = 0.0002; MD = −2.61; 95% CI −4.00 to −1.23; WBFSnurses *p* = 0.0001; MD = −2.71; 95% CI −2.82 to −2.60; VAS/NRS *p* = 0.001, MD = −0.71; 95% CI −1.13 to −0.28), anxiety (CAMpatient *p* = 0.02, MD = −2.92; 95% CI −5.45 to −0.38; CAMparents *p* = 0.01, MD = −3.87; 95% CI −6.99 to −0.75) and fear (CFSpatients *p* = 0.0005, MD = −1.27; 95% CI −1.99 to −0.56; CFSparents *p* = 0.0005, MD = −1.33; 95% CI −2.08 to −0.58; and CFSnurses *p* = 0.04, MD = −1.13; 95% CI −2.24 to −0.03). However, high heterogeneity was noted. The use of virtual reality as a distraction appears to be a valuable strategy for reducing pain, fear, and anxiety during needle-related procedures, although further studies with higher methodological rigor, based on a standardized protocol, are needed.

## 1. Introduction

During healthcare for children and adolescents, various invasive procedures are performed, which involve techniques carried out by a healthcare professional, often the nurse, wherein the body is chemically and/or mechanically assaulted by introducing a medical device into the body through the skin or an orifice [1]. The most frequently and routinely performed invasive procedures in hospitals and healthcare centers are needle-related procedures, such as intravenous cannulation and phlebotomy. They are identified as the major sources of pain, fear, and anxiety in children and adolescents [2], who tend to understand the necessity of the intervention but not that the procedure should cause pain [3]. In this regard, concerning prevalence, a recent survey conducted among a population including European children and adolescents found that 60.1% and 48.1% rated pain, fear, and anxiety as the factors that most concerned them about their healthcare [4].

The International Association for the Study of Pain defined pain as an “unpleasant sensory and emotional experience associated with, or resembling that associated with, actual or potential tissue damage” [5]. The assessment and treatment of pain in children and adolescents is one of the primary objectives of nursing care when performing invasive needle-related procedures [6].

According to the DSM-V, fear is an emotional response to an imminent threat, either real or imagined, while anxiety is an anticipatory response to a future threat. Specifically, anxiety is defined as the anticipation of a future threat arising from the perception of potentially harmful general stimuli, evoking a state of restlessness, agitation, worry, and hypervigilance [7].

Due to the memories generated by these experiences in childhood and adolescence, negative associations with these same techniques can be established, affecting later stages of life as people grow [8]. Therefore, these feelings can lead to avoidant behavior in children and adolescents toward subsequent procedures such as vaccination or other needle-related procedures. This can translate into a needle phobia in 10% of adults, due to negative past experiences as children and/or adolescents [4].

Authors like Eijlers et al. (2019) conclude in their work that the anticipatory fear of invasive procedures contributes to a greater intensity of pain and anxiety in subsequent interventions, thus creating an escalating cycle of pain and distress [9]. However, despite its high prevalence, clinicians in clinical settings have always tended to use pharmacological measures to alleviate procedural pain and stress. While drugs can quickly relieve pain, they can also generate adverse effects, making non-pharmacological interventions a potentially safer method to alleviate pain and negative emotions [10].

Distraction is a non-pharmacological strategy used by nurses during invasive procedures in children and adolescents to manage pain, fear, and anxiety by diverting their attention from nociceptive stimuli, thereby reducing their awareness of pain [11]. In this regard, for a distractor to be effective, it must stimulate the senses, be developmentally appropriate, and be highly interactive to capture the child’s or adolescent’s attention [12]. Therefore, it is vital to find new techniques to reduce pain, fear, and anxiety in pediatric patients.

In recent years, there has been growing interest in the use of virtual reality (VR) as a non-pharmacological distractor for controlling pain, anxiety, and fear during invasive procedures, by creating a three-dimensional artificial environment that engages the visual, auditory, and proprioceptive senses. Additionally, VR goggles block the wearer’s view of the users of the physical environment, reducing anticipatory fear of the procedure [13].

VR is defined as an artificial environment experienced through audiovisual stimuli provided by a computer [14]. It immerses users in a 360° three-dimensional alternative reality by using a headset with motion sensor goggles, and in the case of immersive VR, users can interact with the environment through a handheld controller [15,16]. Until a few years ago, the use of VR was not feasible due to its high cost and complexity of use, but new generations of VR devices are more affordable and easier to use, making VR an interesting and innovative tool [17].

Therefore, the objective of this study is to evaluate the effectiveness of VR as a distraction measure to reduce pain, fear, and anxiety in children and adolescents undergoing needle-related invasive procedures.

## 2. Materials and Methods

### 2.1. Search Strategy and Inclusion Criteria

An exhaustive search was conducted in the following databases: PubMed, CINAHL (Cumulative Index to Nursing and Allied Health Literature), Scopus, Web of Science, and the Cochrane Library. PRISMA (Preferred Reporting Items for Systematic Reviews and Meta-Analyses for Protocols) and AMSTAR-2 (a measurement tool to assess systematic reviews) were followed [18,19]. To confirm the absence of similar documents, a search on ClinicalTrials.gov and in the PROSPERO (Prospective Register of Systematic Reviews) registry was carried out. Google Scholar was also explored to minimize potential publication biases. The protocol for this study was registered on the PROSPERO website with the code CRD42024563245.

The search terms used were (child* OR adolescen* OR youth* OR “young person” OR teen OR pediatric* OR pediatric*) AND (pain* OR anxiety OR fear) AND (“medical procedure” OR “invasive procedure” OR procedure* OR “interventions”) AND (“VR” OR “virtual reality” OR “virtual reality distraction”) and their equivalent in Spanish, French, or Italian. 

These search terms were obtained from the medical subject headings (MeSH) and were used during the search, which was conducted from January to May 2024 by two researchers independently (M.C.-G. and E.M.). Inclusion criteria comprised articles published from 2014 to 2024, in English or Spanish, concerning children and/or adolescents (4–22 years old), on topics related to the objectives of this study, and randomized clinical trials (RCTs) conducted in adult humans. The exclusion criteria were included to consider the assessment of the methodological quality of the included studies to mitigate biases. Studies related to central catheter punctures, such as reservoirs, lumbar punctures, dental procedures, and pain management in other contexts, such as burn treatment or injury management, were also excluded.

To systematize the screening process, an Excel spreadsheet (version Office Profes-sional Plus 2016 for Windows (Microsoft, Redmond, WA, USA)) was created with the selected articles, on which two authors independently assessed their suitability, categorizing them as accepted, rejected, or uncertain. In case of disagreement regarding the inclusion or exclusion of a study, a third investigator (R.C.-M.) was consulted.

### 2.2. Data Extraction 

The search and the article selection were independently conducted by two researchers (M.C.-G. and E.M.), and, in case of disagreement, the opinion of an expert in chronic pain was considered for resolution (R.C.-M.). Data extraction for the meta-analysis was also conducted independently and systematically by two researchers (M.P.-C. and R.C.-M.).

Initially, the titles and abstracts of the articles were reviewed, followed by a full assessment of the selected articles. Additionally, a bibliographic search was conducted both forward and backward in the references cited in the selected studies. The agreement between the two researchers in assessing the suitability of the studies was quantified using the Kappa statistic. 

A data coding manual was used to collect information from each study, including: (1) the author’s name; (2) the year of publication; (3) the country of origin; (4) the sample size (disaggregated by sex and with the average age); (5) the type of intervention (VR use versus the control group); (6) measurement instruments/time points; (7) findings for the experimental and control groups; and (8) final outcomes. The primary continuous outcomes analyzed included pain, measured using the Wong–Baker faces scale (WBFS) [20] as reported by patients, parents, and healthcare professionals, as well as the numerical rating scale (NRS) [21]; anxiety levels, measured using the children’s anxiety meter (CAM) [22] as reported by parents and healthcare professionals; fear, assessed with the children’s fear scale (CFS) [23] reported by children and/or adolescents, parents, and healthcare professionals; intervention time in minutes; and finally, the satisfaction levels of staff, patients, and healthcare providers.

### 2.3. Quality and Bias Risk Assessment

The risk of bias in the included clinical trials was independently assessed in duplicate by two researchers (M.C.-G. and E.M.) using the Cochrane Risk of Bias tool (version 2.0 (Cochrane, London, UK)) [24]. The studies were evaluated across six domains: randomization and sequence generation, allocation concealment, blinding of outcome assessment, completeness of outcome data, selective outcome reporting, and ethical concerns. Additionally, factors such as conflicts of interest with commercial entities and small sample sizes were considered. Due to the nature of the intervention, RCTs that could not implement the blinding of personnel and participants were not penalized, as this is often infeasible with many instruments.

Each risk type was categorized into three levels: low, high, or unclear. Studies with no high risk of bias in any category were considered high quality (1++), while those with a high risk or two unclear risks were rated as moderate quality (1+). All other studies were deemed low quality (1−).

The methodological quality of the RCTs was also assessed using the modified Jadad scale, with a score of ≥ 4 indicating high quality. Additionally, data were imported into the GradePro application (version GradePro GDT (McMaster University and Evidence Prime Inc., Hamilton, Canada)) to evaluate the strength of the recommendations for the obtained results [25].

### 2.4. Data Synthesis and Statistical Analysis 

Continuous variables were evaluated using mean differences (MD) accompanied by a 95% confidence interval (CI). The results were pooled, based on the same measurement scale. In cases where standard deviation data were unavailable, the method recommended by Hozo et al. [26] was employed. The data were analyzed using either fixed-effects or random-effects models. Initially, the fixed-effects model was selected when there was no significant heterogeneity among the studies (I^2^ ≤ 50%). If significant heterogeneity was present, the random-effects model was applied. For the NRS variable, standardized mean differences (SDM) were assessed, along with a 95% CI, due to the heterogeneity of the measure.

Heterogeneity among the studies was assessed using chi-square tests and the I^2^ statistic, with a significance level set at a *p*-value of < 0.05. I^2^ values between 0% and 25% indicated low heterogeneity, values between 25% and 75% indicated moderate heterogeneity, and values above 75% indicated high heterogeneity [27].

A forest plot was used to visualize the results of the meta-analysis, and a funnel plot was employed to evaluate potential publication bias among the studies. The asymmetry of the funnel plot was analyzed visually and assessed with an Egger’s test [28], with a *p*-value of <0.05 considered to be indicative of evidence of publication bias.

A sensitivity analysis was also conducted to assess the robustness of the results by sequentially excluding each study; *p*-values of <0.05 were considered to be statistically significant.

Statistical analysis and bias assessment were performed using Review Manager software, version 5.4 (Cochrane Library, London, UK). 

## 3. Results

### 3.1. Results Obtained in the Selection of Articles

In the initial literature search, 1477 articles were identified. No additional documents were excluded from specific clinical trial registries such as ClinicalTrials.gov and PROSPERO. After removing 806 duplicate manuscripts using the Zotero^®^ reference manager (version 7.0 (George Mason University, Fairfax, VA, USA)) and applying the inclusion criteria to assess titles and abstracts, 611 articles were excluded due to their not meeting the established criteria. Ultimately, 21 studies were selected for the systematic review, of which 18 provided data for the meta-analysis, encompassing a total sample of 2663 participants, who were subjected to a distraction intervention with VR (n = 1145 in the VR experimental group vs. n = 1095 in the standard care control group), plus 423 participants who underwent other distraction techniques such as the cold vibration method or the use of playground equipment.

**Figure 1:**
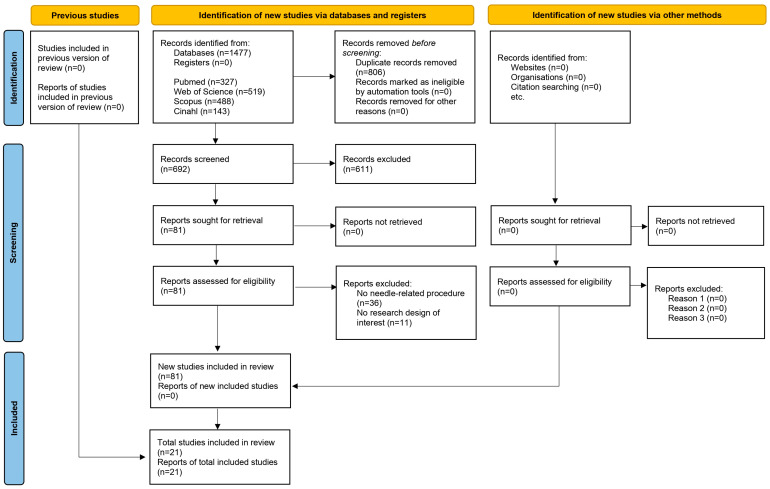
Flow diagram that illustrates the review process.

The flow diagram (Figure 1) illustrates the review process. The agreement between the researchers in assessing the eligibility of the trials was excellent (Kappa statistic = 0.90).

### 3.2. Descriptive Analysis of the Results Found

Of the 21 clinical trials included in the systematic review, 100% (n = 21) were randomized; no crossover trials were found (0.0%). Regarding the distribution by year, there was one trial each in 2017 (n = 1; 4.8%), 2018 (n = 1; 4.8%), and 2024 (n = 1; 4.8%); two trials each in 2020 (n = 2; 9.5%) and 2021 (n = 2; 9.5%); three trials in 2022 (n = 3; 14.3%); and the highest number of articles were from 2019 (n = 5; 23.8%) and 2023 (n = 6; 28.6%).

The topics studied included: pain (n = 21); anxiety (n = 11); fear (n = 7); procedural time (n = 8); and satisfaction level (n = 5). When analyzing the scales used for pain, the following were observed: NRS (n = 4), VAS (n = 6), WBFS (n = 9), CAS-C (n = 2), and FPS-R (n = 6). For anxiety, the scales used were CAM-S (n = 3), CSAS-C (n = 1), VAS (n = 3), VSA (n = 1), FAS (n = 1), GDS (n = 1), and STAIC (n = 1). Finally, fear was assessed using the CFS scale (n = 5) and CMFS (n = 1). In contrast, process duration was evaluated in 100% of the cases in minutes, and satisfaction level was measured on a scale from 0 to 10, where 0 was considered “not satisfactory” and 10 was “the highest level of satisfaction”.

Regarding the assessment of measurements, 38.0% (n = 8) conducted only a post-intervention assessment. A total of 52.4% (n = 11) performed both pre- and post-intervention assessments. One study (n = 1; 4.8%) conducted assessments at three time points: before, during, and after the process. Finally, one study (n = 1; 4.8%) performed assessments at three time points: before and after the intervention, and at 30 min post-intervention.

Regarding the study age range, there is considerable heterogeneity among the age intervals, with the following groups observed: 4–6 years (n = 1; 4.8%); 4–7 years (n = 1; 4.8%); 4–10 years (n = 1; 4.8%); 4–11 years (n = 1; 4.8%); 4–12 years (n = 1; 4.8%); 5–9 years (n = 1; 4.8%); 5–12 years (n = 1; 4.8%); 6–12 years (n = 1; 4.8%); 6–16 years (n = 1; 4.8%); 6–18 years (n = 1; 4.8%); 7–11 years (n = 1; 4.8%); 7–12 years (n = 4; 19.0%); 7–16 years (n = 1; 4.8%); 7–17 years (n = 1; 4.8%); 8–17 years (n = 1; 4.8%); 9–12 years (n = 1; 4.8%); 10–21 years (n = 1; 4.8%); and 12–17 years (n = 1; 4.8%). Indeed, clinical trials on the use of VR have increased in recent years. As we can detect from the Pubmed database, until 2018, no more than 3 clinical trials had been published each year. Then, in 2019, the scientific production of clinical trials grew enormously, going from 1 (2018) to 5. For this reason, we considered it appropriate to highlight this data. Regarding the distribution by year, there was an increase from 2019 until 2024, with 19 (90.47%) being the number of articles published in this period. The highest numbers of articles were from 2019 (n = 5; 23.8%) and 2023 (n = 6; 28.6%). 

The levels of evidence, assessed according to the quality of the selected articles, received the following ratings: 1++ (n = 6; 28.6%), 1+ (n = 11; 52.4%), and 1− (n = 4; 19.0%). Details of each included article can be found in Table 1.

### 3.3. Assessment of Risk of Bias in Selected Studies and Publication Bias

**Figure 2:**
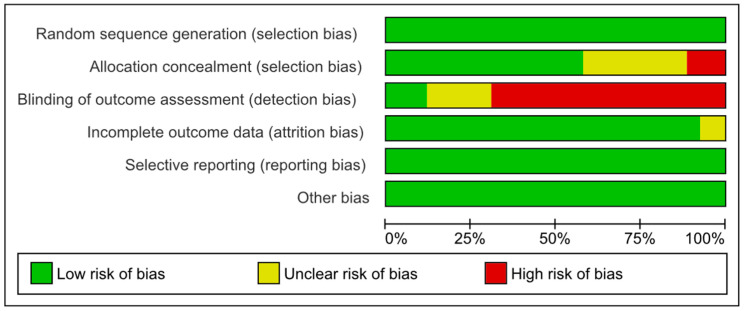
Risk of bias graph: review of authors’ judgments about each risk of bias item, presented as percentages across all included studies. Red = high risk; green = low risk; yellow = unclear risk; +/− = risk percentage.

**Figure 3:**
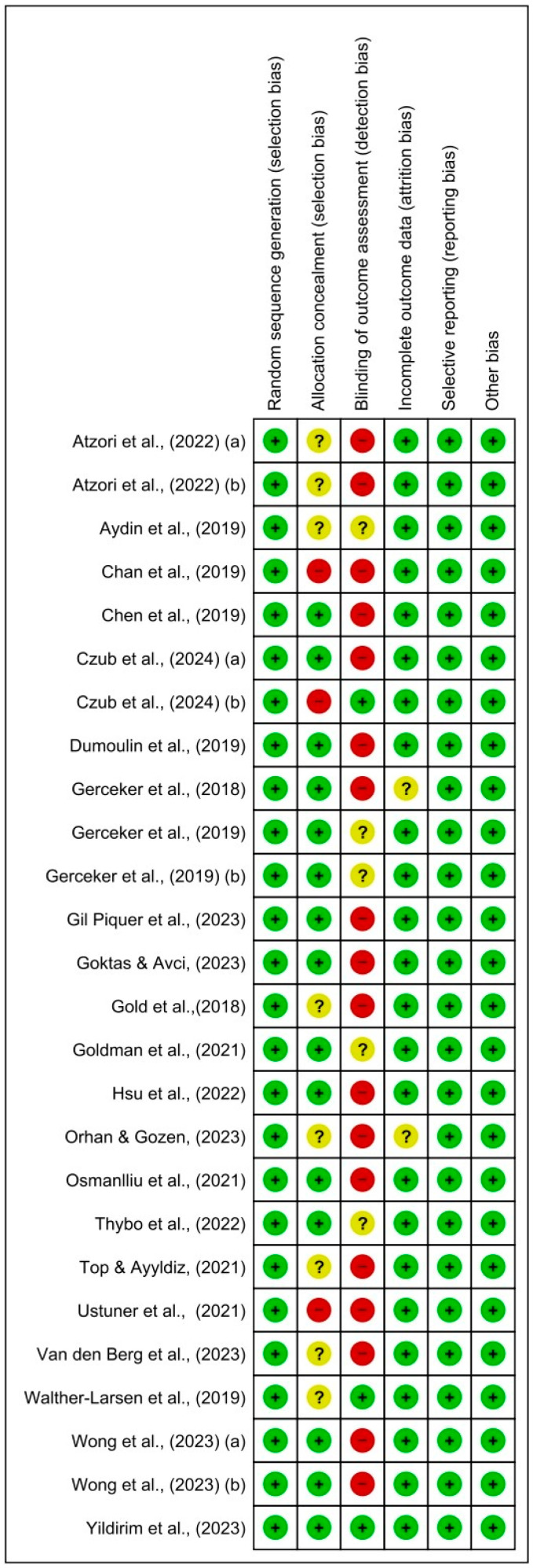
Risk of bias summary: review authors’ judgments about each risk of bias item for each included study. Red = high risk; green = low risk; yellow/? = unclear risk; +/− = risk percentage.

Bias risk was assessed using RevMan5^®^, and the bias assessment graphs are presented in Figure 2 and Figure 3, showing both a general evaluation of all included studies and an individual evaluation of each study. In 35% of cases, allocation concealment was unclear. Approximately 15% of cases had blinding in the outcome assessment, and random sequence generation was 100% in all cases. To assess publication bias, Egger’s test was used. It was estimated that, apart from satisfaction levels (*p* = 0.03), the remaining study variables did not show significant evidence of publication bias (*p* = 0.05).

The level of recommendation was determined using GradePro after exporting the results from RevMan5^®^. A recommendation grade between low and high was obtained for the use of virtual reality regarding pain, depending on the scale used, with a recommendation grade of low for anxiety, and a recommendation grade of low for fear management (Table 2).

### 3.4. Results of Meta-Analysis

#### 3.4.1. Assessment of Pain Using Virtual Reality

##### Use of the WBFS Scale

**Figure 4:**
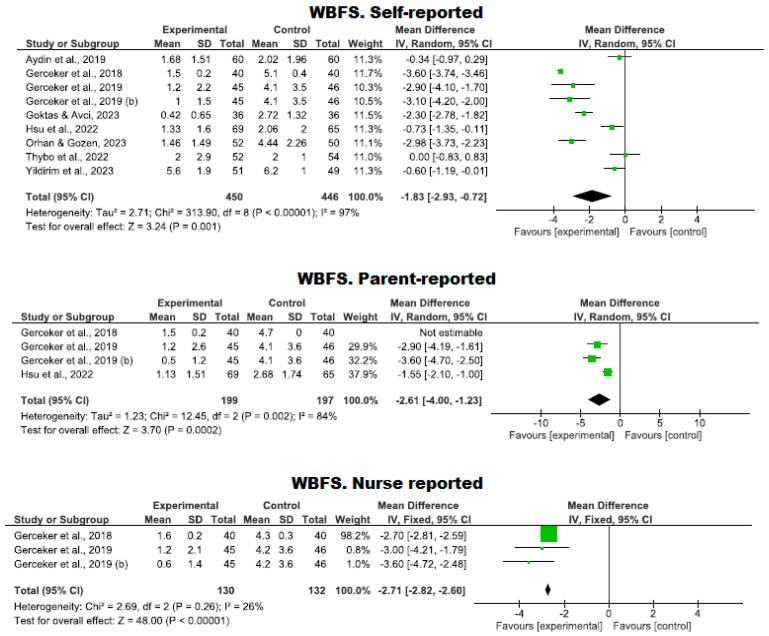
Pain evaluation using the WBFS scale by the patient, parents, and nursing staff.

In nine clinical trials involving 896 participants (450 in the intervention group and 446 in the control group), the efficacy of using VR to reduce pain was compared using the WBFS scale against standard care. One study showed a high risk of bias [39], while two exhibited an adequate quality level [29,33], and five met a high-quality standard [10,34,36,41,42] (Figure 4).

A lower level of pain was observed in the VR group compared to the control group, as reported by the patients themselves (*p* = 0.001; MD = −1.83; with a 95% CI −2.93 to −0.72), by the parents (*p* = 0.0002; MD = −2.61; with a 95% CI −4.00 to −1.23), and by the nursing staff (*p* = 0.0001; MD = −2.71; with a 95% CI −2.82 to −2.60). However, high heterogeneity was observed in the first two reports (patients and parents) (I^2^ = 97% and 84%, respectively). The fixed-effect model was used only for nurse-reported pain.

##### Use of the NRS and VAS Scales

Similarly, pain was assessed using two scales with comparable units: the NRS and VAS scales. Although a direct comparison of both measurements is possible, the standardized mean difference (SMD) was used.

**Figure 5:**
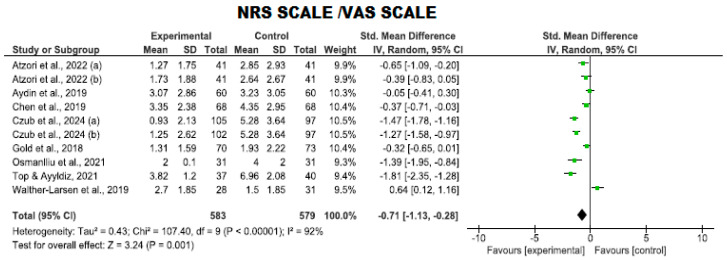
Pain evaluation using the NRS and VAS scale.

In this analysis, eight clinical trials involving 1162 participants (583 in the intervention group and 579 in the control group) were evaluated to compare the efficacy of VR in reducing pain using the aforementioned scales against standard care. No trials were found to have a high risk of bias, while six exhibited an adequate quality level [8,29,30,32,37,40], and two met a high-quality standard [41,43] (Figure 5).

A lower level of pain was observed in the VR group compared to the control group (*p* = 0.001, MD = −0.71; with a 95% CI −1.13 to −0.28). However, high heterogeneity was noted (I^2^ = 92%).

##### Other Pain Assessment Scales

With regard to the assessment of pain with the FPS-R scale, six clinical trials were evaluated [1,12,31,38,39,42]. All of them showed a significant reduction in pain in the experimental group with respect to the control group (*p* < 0.05). In addition, the study by Thybo et al. [38] found that crying time was shorter in the VR group compared to the control group (8.43 ± 12.42 s vs. 33.65 ± 24.02 s).

#### 3.4.2. Assessment of Anxiety Using Virtual Reality

##### Use of the CAM-S Scale

Anxiety was evaluated through three clinical trials involving 424 participants (210 in the intervention group and 214 in the control group). The efficacy of using VR to reduce anxiety was compared using the CAM-S scale against standard care. No studies showed a high risk of bias; one study exhibited an adequate quality level [37], while two met a high-quality standard [34,42].

**Figure 6:**
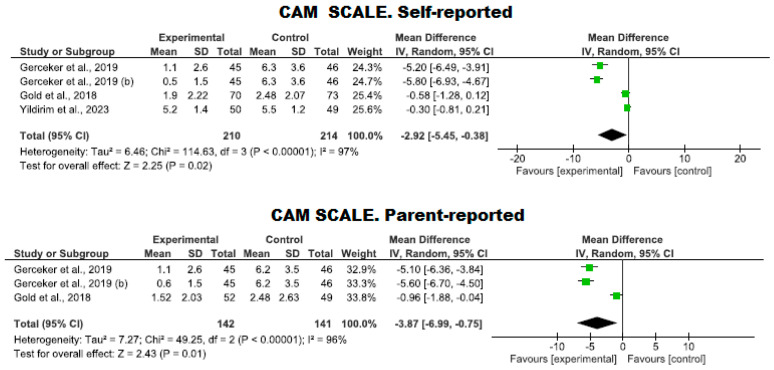
Assessment of anxiety using the CAM-S scale by both the patient and the parents.

A lower level of anxiety was observed in the VR group compared to the control group, as reported by the patients themselves (*p* = 0.02, MD = −2.92; with a 95% CI −5.45 to −0.38), and by the parents (*p* = 0.01, MD = −3.87; with a 95% CI −6.99 to −0.75). However, high heterogeneity was noted (I^2^ = 97% and 96%, respectively) (Figure 6).

##### Use of Other Scales to Assess Anxiety

Similarly, regardless of the scale used, studies suggest that anxiety improves with VR compared to the control group. Scales with similar data to the CAM-S, such as the VAS [31,32,37,38], anxiety-GDS child [35], visual analog scale for anxiety VAT [12], and state-trait anxiety inventory for children (STAIC) [39] have been used, with statistically significant results in favor of VR.

#### 3.4.3. Management of Fear Using Virtual Reality

With regard to the perception of fear, this was evaluated through five clinical trials involving 624 participants (314 in the intervention group and 310 in the control group). The efficacy of using VR to reduce the perception of fear was compared using the CFS scale against standard care. No studies showed a high risk of bias; one study exhibited an adequate quality level [30], while four met a high-quality standard [10,34,36,42].

**Figure 7:**
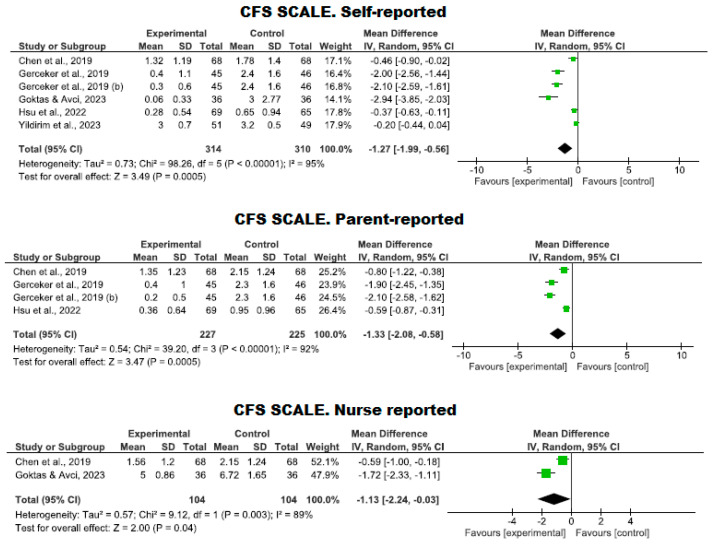
Assessment of fear using the CFS scale by the patient, parents, and nursing staff.

A lower level of fear was observed in the VR group compared to the control group, as reported by the patients themselves (*p* = 0.0005, MD = −1.27; with a 95% CI −1.99 to −0.56), by the parents (*p* = 0.0005, MD = −1.33; with a 95% CI −2.08 to −0.58), and by the nursing staff (*p* = 0.04, MD = −1.13; with a 95% CI −2.24 to −0.03). However, high heterogeneity was noted in all reports (I^2^ = 95%, 92%, and 89%, respectively) (Figure 7).

#### 3.4.4. Procedural Time Using Virtual Reality

**Figure 8:**
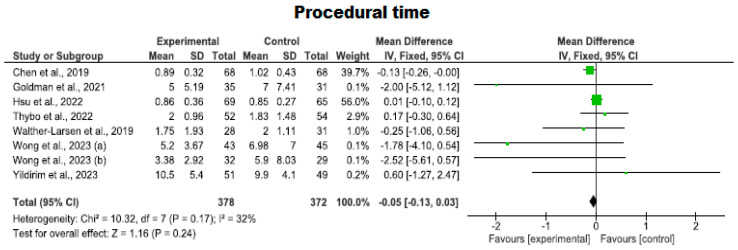
Assessment of the duration of the procedure (minutes).

With regard to procedure time, seven clinical trials involving 750 participants (378 in the intervention group and 372 in the control group) were analyzed. The duration of procedures involving needles was compared when using VR against standard care. No studies showed a high risk of bias; two studies exhibited an adequate quality level [30,43], while five met a high-quality standard [1,10,38,41,42] (Figure 8).

A slight trend toward longer procedure times was observed in the VR group compared to the control group; however, the findings were inconclusive as the results approached the line of no effect (*p* = 0.24, MD = −0.05; with a 95% CI −0.13 to 0.03). Low heterogeneity was observed (I^2^ = 32%). Therefore, the fixed-effect model was used.

#### 3.4.5. Degree of Satisfaction with the Use of Virtual Reality

Finally, we analyzed satisfaction in five clinical trials with 777 participants, comprising 393 in the intervention group and 384 in the control group, assessing satisfaction with VR use to cope with needle-based procedures compared to standard care. No studies with a high risk of bias were found, while three exhibited an adequate level of quality [31,40,43] and two met a high level of quality [1,41].

A higher level of satisfaction was observed in the VR group compared to the control group with respect to providers (*p* = 0.0007 MD = 1.01; 95% CI 0.43 to 1.59). No such findings were found when patients and parents were asked (*p* = 0.88 MD = −0.05; 95% CI −0.76 to 0.65). Specifically, in the patient group, only a single study could be evaluated, making comparisons and, thus, results impossible. Heterogeneity was high (I^2^ = 84% and 91%).

**Figure 9:**
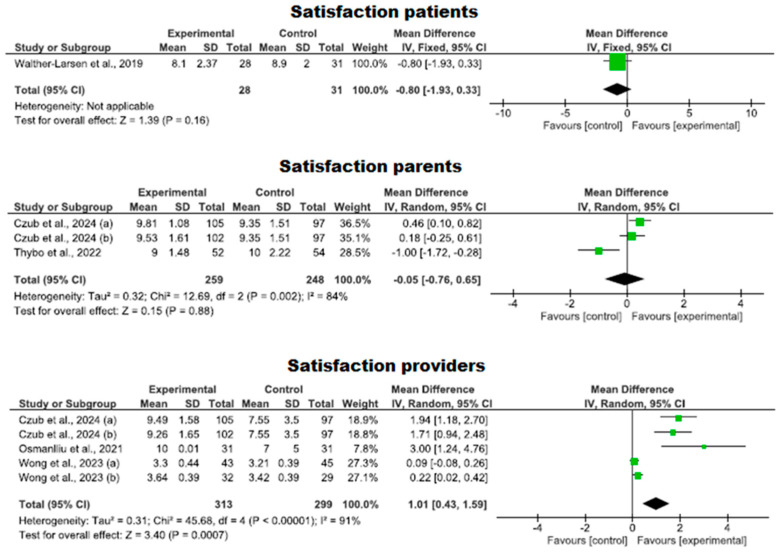
Levels of satisfaction with VR among patients, parents, and providers.

Along the same lines, there are studies that evaluate the degree of satisfaction of children, who consider the use of VR to be significantly more “*fun*” than the use of standard procedures (*p* < 0.024) [38] (Figure 9).

## 4. Discussion

This meta-analysis is the most current to date to evaluate the effectiveness of VR as a distraction measure to reduce pain, fear, and anxiety in children and adolescents undergoing needle-related invasive procedures. Outcomes were assessed in terms of pain intensity after procedures; level of anxiety and fear after procedures; and procedural time and degree of satisfaction with VR. The analyses of the scales used for pain assessment showed the following distribution: NRS (n = 4), VAS (n = 6), WBFS (n = 9), CAS-C (n = 2), and FPS-R (n = 6). To assess anxiety, the scales used were CAM-S (n = 3), CSAS-C (n = 1), VAS (n = 3), VSA (n = 1), FAS (n = 1), GDS (n = 1), and STAIC (n = 1). Finally, fear was assessed using the CFS scale (n = 5) and CMFS (n = 1).

Despite the considerable number of RCTs included in this meta-analysis, the results do not provide enough evidence to determine the effectiveness of VR in reducing pain, anxiety, and fear during needle-related procedures when compared with standard care interventions, due to high heterogeneity between the included studies (I^2^ > 20%). Nevertheless, given that this meta-analysis was conducted on pediatric and adolescent populations, and that this age range is very broad, it is not possible to determine the influence of age on the effectiveness of virtual reality as a distractor.

In this study, an effort was made to homogenize and classify by age in order to conduct the meta-analysis separately, but this proved impossible because the original RCTs did not differentiate by age group. It is necessary to consider that the indications for the use of these types of VR electronic devices advise against their use in children under 13 years of age [44]. However, many of the age groups of the participants included in the RCTs are younger than this age in many cases. This has also occurred in previous meta-analyses with similar objectives to this study and in the same population [43,45,46].

However, a positive tendency in the use of VR in acute pain control, anxiety, and fear after procedures, as well as in the level of satisfaction in healthcare providers, in comparison with control groups was observed, obtaining a statistically significant association (*p* < 0.05). This could indicate that the use of VR is more effective for these outcomes. Conversely, no statistically significant association was found between the degree of satisfaction in parents (*p* = 0.88) and procedural time in minutes (*p* = 0.24).

Regarding the evaluation of the effectiveness of VR in controlling acute pain intensity post-intervention, the included studies have used various scales, such as the WBFS, VAS, and NRS. This diversity complicates the comparison of VR effectiveness across all studies. However, authors like Gao et al. (2022) do not differentiate between the WBFS, VAS, and NRS scales in their study [43]. This study, in addition to including more recent RCTs, has considered a more homogeneous approach to the measurement scales. Nevertheless, in general terms, the findings obtained in the meta-analyses by Gao et al. (2022) and Czech et al. (2021) align with the findings of this study [43,45].

Another aspect to consider, once again, is age, as numerous myths have traditionally affected the management of acute pain in this population by healthcare professionals, especially in children, who experience pain differently from adolescents. Notable among these myths is the belief that children lack the capacity to perceive painful stimuli or to remember them over time [47]. When pain and the associated anxiety and fear are not effectively managed, post-traumatic stress symptoms may occur in children and adolescents, causing a negative attitude toward needle-related procedures in the future [48,49].

This issue has been mitigated through the use of topical medications such as EMLA^®^ (AstraZeneca, Cambridge, UK) anesthetic cream (25 mg/g lidocaine + 25 mg/g prilocaine) or *Cloretilo Chemirosa*^®^ (ERN, Barcelona, Spain) spray (ethyl chloride 100 g). The use of this medication is not without controversy, as it has the capacity to change the depolarization of the cell membrane to sodium ions, thereby blocking the conduction of nerve impulses that can cause pain in the superficial layers of the skin (up to 5 mm in depth) [50]. In a study carried out by Moore et al. (2013), the authors stated that when patients received a VR intervention, the activity of neuroanatomical pain matrix regions decreased by more than 50% [51].

Triberti et al. (2014) added that its ability to distract has the greatest effect on pain reduction and on the control of anxiety and fear after needle-related procedures [52]. According to a systematic review conducted by Uman et al. (2013), other distraction strategies for controlling pain, fear, and anxiety include hypnosis, distraction by parents, and breathing tasks. Other techniques were found to be ineffective for controlling fear and anxiety in the review mentioned [53].

With regard to anxiety and fear, both feelings cannot be separated from pain. It is known that high levels of fear and anxiety increase pain levels and vice versa [54]. The results of this study suggest a greater effectiveness of VR in managing stress and anxiety compared to the control group, findings that are consistent with those of Gao et al. (2022) and Czech et al. (2021) [43,45].

In terms of differences between the two articles, it should be noted that the meta-analyses conducted by Jenabi et al. (2023) [46] and Gao et al. (2023) [43], despite being published in 2023, only included clinical trials up to the years 2021 and 2022, respectively, while the present work includes recently published studies. In contrast, the review by Gao et al. (2023) only includes pain assessment [43], whereas the review by Jenabi et al. (2023) included other aspects such as anxiety. However, this review included additional aspects such as the evaluation of the time of needle-related procedures, user satisfaction, and feedback from parents and professionals, among others, providing a broader perspective on the effectiveness of virtual reality [46].

Regarding healthcare professionals’ satisfaction and the duration of the procedure, the results showed greater satisfaction reported in the group that utilized VR compared to the control group. However, the average procedural time was longer in the experimental group in the majority of the RCTs included in this study. Furthermore, once professionals become familiar with the devices and with the cooperation of children and adolescents, it is possible that the time required to perform the procedure could be reduced, along with a decrease in the material resources needed.

From a healthcare resource management perspective, the use of VR to reduce pain, fear, and anxiety in children and adolescents undergoing needle-related procedures can entail significant healthcare costs but can also present long-term economic benefits [54]. Initially, the acquisition and maintenance of VR equipment can represent a substantial investment. However, these costs can be offset by reducing the need for topical analgesics and potential adverse events or side effects. Studies have demonstrated that more immersive VR systems are increasingly accessible and affordable, facilitating their implementation in clinical settings. The expense associated with implementing virtual reality (VR) in managing pain, fear, and anxiety during medical procedures for children and adolescents can be significant, but its value should be evaluated in the context of potential benefits and long-term savings [55,56].

Advanced VR systems, such as the Oculus Rift and HTC Vive, require high-end computer equipment or dedicated gaming consoles, with costs that can exceed several hundred dollars per unit [56]. However, more accessible options, such as smartphone-based systems like Google Cardboard, use mobile devices to provide a VR experience at a much lower cost [57]. Although the initial investment in VR technology can be substantial, its use in clinical settings offers the potential to reduce reliance on pharmacological interventions and decrease the overall time of procedures, which can translate into cost savings in the long term.

VR has proven effective in reducing pain and anxiety during invasive procedures, which can minimize the need for additional treatments and enhance the patient experience [58]. Furthermore, the use of VR may lead to greater adherence to procedures and improve overall clinical outcomes, supporting investment in these technologies. Research has demonstrated that virtual reality not only reduces the perception of pain and discomfort but also has the potential to decrease the number of failed interventions and enhance the efficiency of medical procedures [59]. Thus, although the initial cost of VR systems may appear high, the benefits in terms of pain reduction, anxiety alleviation, and savings on additional treatment costs justify the investment, promoting the adoption of VR in pediatric settings for a more effective and less invasive pain management approach.

### 4.1. Limitations and Strengths

A thorough analysis of the literature was conducted to provide the most up-to-date evidence on the use of VR to mitigate the negative effects of venipuncture. Despite the merits of this study, it is not without limitations, which are described below.

Firstly, there are challenges in homogenizing the ages of participants, making it impossible to distinguish between children and adolescents. This situation may affect the external validity of the results, as the response level of a child to such a situation differs from that of an adolescent, who presumably has greater competencies in managing pain and emotions. Additionally, the heterogeneity in the use of scales and devices complicates the unification of criteria, although almost all point to a substantial improvement compared to the control group. Some authors, such as Czub et al. [30], even indicate a high correlation between the different scales, suggesting that this variable does not significantly affect the results.

Furthermore, other factors related to the heterogeneity of the results may exist, beyond the differences in age groups and measurement instruments used. These include the lack of standardization in the types of VR devices, the experience of the professionals performing the technique and measuring the outcomes, the variation in the time elapsed between the procedure and the evaluation of the outcomes, and sociocultural differences among the participants of the various studies.

There are also gaps in some measured variables, such as the satisfaction levels of nursing staff with the use of these devices. Finally, it must be considered that, although patients with reservoirs are excluded, the procedures evaluated in this manuscript include interventions ranging from blood extraction to the cannulation of peripheral venous access. These situations may influence the pain experienced and the time required to complete the procedure, as well as other related variables.

Finally, articles published from 2014 onward were used as the limit, which, although there has been an increase in scientific production since then, needs to be considered as a limitation because some articles may have been missed in the process.

Regarding strengths, this meta-analysis evaluates more outcomes than previous meta-analyses and includes studies published in the current year.

### 4.2. Prospective Lines

The implications of this meta-analysis for nursing practice could facilitate the integration of this technology as an effective tool, offering an innovative alternative to reduce the discomfort associated with these procedures. This may lead to improvements in pain and anxiety management protocols and the optimization of resources by reducing the need for additional sedation. Furthermore, implementing these technologies will require appropriate training for nursing staff and education for patients and families, which can also enhance the patient’s experience and provide more personalized care. The evidence derived from these studies can inform the development of clinical guidelines and evidence-based protocols, thereby updating practices and ensuring patient-centered care. Finally, the results of the meta-analysis may justify investment in virtual reality technologies, highlighting their potential to improve the quality of care and outcomes in pediatric patients.

It is recommended to increase the number of clinical trials that emphasize methodological improvements, particularly regarding the blinding of evaluators and the concealed allocation of participants. Additionally, it would be beneficial to disaggregate the sample into children and adolescents, to determine if there are differences between these age groups, as well as to standardize the most relevant scales for measuring pain, anxiety, and fear in this population.

Moreover, it would be worthwhile to compare VR with other distraction techniques through cost-effectiveness studies to determine the real benefits of VR relative to its cost compared to other less expensive devices, such as the use of toys with medical equipment or vibration with cold using the Buzzy device, among others.

## 5. Conclusions

The use of VR as a distraction method for children and adolescents appears to be a valuable strategy for reducing pain, fear, and anxiety during needle-related procedures, as well as for improving the experience for parents and healthcare providers. However, the findings do not provide conclusive results regarding the degree of satisfaction and procedural time. Further studies with higher methodological rigor, based on a standardized protocol, are needed.

## Figures and Tables

**Table 1:** Characteristics of the Included studies.

Study/Country /Procedure/Setting/ Level of Evidence	Design/Sample (Gender/Mean Age ± SD, Years or Median with IQR)	Intervention (During the Procedure)	Outcomes/Instruments/Measurement’s Time	Experimental Group(s) Finding (Mean ± SD or Mean Difference or CI)	Control Group(s) Finding (Mean ± SD or Mean Difference or CI)	*p*-Value Main Finding
Atzori et al. [8] (2022)/Italy VENIPUNCTURE (Children’s hospital) 1+	RCT n = 82 VRG = 41 (20 F, 21 M; 11.39 ± 2.73) CG = 41 (18 F, 23 M; 12.17 ± 2.62)	VRG: immersive VR with Snow World software, using VR equipment consisting of a VR helmet and the personal 3D viewer Sony: HMZ T-2, supported by a laptop CG: standard treatment as usual	Pain (worst and emotional): NRS Post-intervention	Pain- NRS 1.56 ± 1.83 (Worst) 1.17 ± 1.80 (Emotional)	Pain-NRS 2.74 ± 2.76 (Worst) 2.41 ± 2.94 (Emotional)	VRG vs. CG Pain (VNRS) <0.05 (Worst) <0.05 (Emotional)
Aydin and Özyazıcıoğlu [29] (2019)/USA VENIPUNCTURE (Clinic) 1+	RCT n = 67 VRG = 60 (30 F, 30 M; 10.50 ± 1.14) CG = 60 (29 F, 31 M; 10.30 ± 12)	VRG: 3D ‘‘Aquarium VR’’ application (simulates a submarine journey to discover things underlying the virtual aquarium) via a virtual reality headset CG: no interventional procedure	Pain: VAS, WBSPF Post-intervention	Pain -VAS: 3.07 ± 2.86 WBSPF: 1.68 ± 1.51	Pain-VAS: 2.02 ± 1.96 WBSPF: 3.23 ± 3.05	VRG vs. CG Pain (VAS, WBSPF) 0.006, 0.039
Chan et al. [12] (2019)/Australia VENIPUNCTURE INTRAVENOUS CANNULATION (Emergency department) 1−	RCT Emergency department study: n = 123 VRG = 64 (29 F, 35M; 7.9 ± 1.5), CG = 59 (27 F, 32 M; 8.2 ± 2.4)	VRG: virtual reality sequence that consists of an interactive underwater adventure, using the “Google Pixel XL” device CG: standard care	Pain: FPS-R Anxiety: visual analog thermometer Child distress (caregiver’s rating): VAS Post-intervention	Pain-FPS-R: −1.39 (−2.42 to –0.36) Anxiety-VAT: −2.2 (−3.20 to –1.20) Child distress (VAS 1 (0–5))	Pain-FPS-R: 0.39 (−1.45 to −0.67) Anxiety-VAT: −0.46 (−1.36 to 0.45) Child distress-VAS: 4 (1–8)	VRG vs. CG Pain (FPS-R): 0.018 Anxiety (VAT): 0.011 Child distress (VAS): 0.004
Chen et al. [13] (2020)/Taiwan INTRAVENOUS INJECTION (Emergency department) 1+	RCT n = 136 VRG = 68 (3 F, 38M; 9.3 ± 1.7) CG = 68 (29 F, 39 M; 9.0 ± 1.7)	VRG: immersive VR using an iPhone device and a head-mounted display (Xiaozhai V4) with four virtual environments CG: regular intravenous injection	Pain: WBSPF Fear: CFS Post-intervention	Pain-WBSPF Child: 3.35 ± 2.38 Primary caregiver: 3.26 ± 2.37 Nurse: 3.29 ± 2.01 Fear-CFS Child: 1.32 ± 1.19 Primary caregiver: 1.35 ± 1.23 Nurse: 1.56 ± 1.20	Pain-WBSPF Child: 4.35 ± 2.95 Primary caregiver: 4.29 ± 2.70 Nurse: 4.29 ± 2.52 Fear-CFS Child: 1.78 ± 1.40 Primary caregiver: 2.03 ± 1.36 Nurse: 2.15 ± 1.24	VRG vs. CG Pain (WBSPF) Child: 0.031 Caregiver:.020 Nurse: 0.12 Fear (CFS) Child: 0.043 Caregiver: 0.003 Nurse: 0.006
Czub et al. [30] (2024)/Poland, Ireland, Spain VENOUS BLOOD DRAW (Pediatric phlebotomy clinics) 1+	RCT n = 312 (304 analyzed) VRG = 105 (39 F, 66 M; 6.8 ± 1.4) GMG = 102 (49 F, 53 M; 6.6 ± 1.3) CG = 97 (50 F, 47 M; 6.6 ± 1.5)	VRG: Magic Spheres game using a Samsung Gear VR system with a Galaxy S8 phone (created by the authors of the study) GMG: two-dimensional version of the Magic Spheres game on a mobile phone screen CG: usual care	Pain: FPS-R, NRS Anxiety: VAS Post-intervention	Pain-FPS-R VRG: 0.933 ± 2.132 GMG: 1.248 ± 2.621 NRS VRG: 1.886 ± 2.411 GMG: 2.110 ± 2.589 Anxiety-VAS VRG: 4.486 ± 3.680 GMG: 4.157 ± 3.506	Pain-FPS-R 3.691 ± 2.132 NRS 5.278 ± 3.63 Anxiety-VAS 3.722 ± 3.744	Pain-(FPS-R) VRG vs. CG: <0.001 VRG vs. GMG: 0.719 GMG vs. CG: 0.001 Correlation FPS-R with NRS: >0.001
Dumoulin et al. [11] (2019)/Canada VENIPUNCTURE IV PLACEMENT (Emergency department of a children’s hospital) 1+	RCT n = 59 VRG = 20 (6 F, 14M; 13.85 ± 2.80) TVCG = 24 (6 F, 9M; 12.65 ± 3.33) CG = 15 (9 F, 9 M; 13.81 ± 2.43)	VRG: immersive real-time game developed by the UQO Cyberpsychology Lab using Virtools 4 TVCG: watch television CG: standard care conditions carried out with the Child Life program intervention	Pain: VAS Fear of pain: VAS Post-intervention	Pain-VAS: 21.75 ± 20.96 Fear of pain-VAS: 19.75 ± 21.18	Pain-VAS TVCG: 35.43 ± 32.65 CG: 25.33 ± 25.25 Fear of pain-VAS TVCG: 35.42 ± 35.93 CG: 29.33 ± 34.48	Pain (VAS) VRG Pre-Post: <0.05 TVCG Pre-Post: <0.05 CG Pre-Post: <0.05 Fear of pain (VAS) VRG Pre-Post: <0.01 TVCG Pre-Post: <0.05 CG Pre-Post: <0.05
Gerçeker.et al. [31] (2018)/Turkey PHLEBOTOMY (Phlebotomy Unit) 1+	RCT n = 147 (121 analyzed) VRG = 40 (20 F, 20 M; 9.7 ± 1.5) BCG = 41(20 F, 21 M; 9.7 ± 1.6) CG = 15 (20 F, 20 M; 8.9 ± 1.3)	VRG: Virtual reality intervention through three videos (Magic English Disney Family, Princess Sofia’s Secret Library, and dinosaur cartoons; PANGEA) using Samsung Gear Oculus with headphones and a Samsung Galaxy S5 Note BCG: Buzzy device with cold and vibration application, starting just before the procedure CG: standard care	Pain: WBSPF Post-intervention	Pain-WBSPF Self-reported: 1.5 ± 0.2 Parent-reported: 1.5 ± 0.2 Nurse-reported: 1.6 ± 0.2 Research-reported: 1.3 ± 0.2	Pain-WBSPF Self-reported BCG: 2.0 ± 0.2 CG: 5.1 ± 0.4 Parent-reported BCG: 2.0 ± 0.2 CG: 4.7 ± 0.4 Nurse-reported BCG: 1.8 ± 0.1 CG: 4.3 ± 0.3 Research-reported BCG: 1.8 ± 0.2 CG: 5.4 ± 0.4	VRG vs. BCG vs. CG Pain (WBSPF) Self-reported, Parent-reported, Nurse-reported, Research-reported: 0.000
Gerçeker et al. [32] (2020)/Turquía BLOOD DRAW (University hospital special draw unit) 1++	RCT n = 141 (136 analyzed) VRRG = 45 (22 F, 23 M; n.d.) VRORG = 45 (21 F, 24 M; n.d.) CG = 46 (20 F, 26 M; n.d.)	VRRG: VR application with a rollercoaster game using a Samsung Galaxy S5 Note mobile phone connected to a virtual headset VRORG: VR application with a submarine experience game, using a Samsung Galaxy S5 Note mobile phone connected to a virtual headset CG: standard care	Pain: WBSPF Fear: CFS Anxiety: CAM-S Post-intervention	Pain-WBSPF Self-reported VRRG: 1.2 ± 2.2 VRORG:1.0 ± 1.5 Parent-reported VRRG: 1.2 ± 2.6 VRORG:0.5 ± 1.2 Nurse-reported VRRG: 1.2 ± 2.1 VRORG: 0.6 ± 1.4 Research-reported VRRG: 1.0 ± 2.2 VRORG: 0.4 ± 1.2 Fear-CFS Self-reported VRRG: 0.4 ± 1.1 VRORG: 0.3 ± 0.6 Parent-reported VRGR: 0.4 ± 1.0 VRGOR: 0.2 ± 0.5 Research-reported VRGR: 0.4 ± 1.0 VRGOR: 0.2 ± 0.5 Anxiety-Self-reported VRGR: 1.1 ± 2.6 VRGOR: 0.5 ± 1.5 Parent-reported VRGR: 1.1 ± 2.6 VRGOR: 0.2 ± 0.5 Research-reported VRGR: 1.0 ± 2.4 VRGOR: 0.6 ± 1.7	Pain-BSPF Self-reported: 4.1 ± 3.5 Parent-reported: 4.1 ± 3.6 Nurse-reported: 4.2 ± 3.6 Research-reported: 4.0 ± 3.6 Fear-CFS Self-reported: 2.4 ± 1.6 Parent-reported: 2.3 ± 1.6 Research-reported: 2.1 ± 1.4/2.3 ± 1.6 Anxiety-CAM-S Self-reported: 6.3 ± 3.6 Parent-reported: 6.2 ± 3.5 Research-reported: 6.3 ± 3.5	VRRG vs. VRORG vs. CG Pain (WBSPF) Self-reported, Parent-reported, Nurse-reported, Research-reported: 0.000 Fear (CFS) Self-reported, Parent-reported, Research-reported VRGR vs. VRGOR vs. CG Post: 0.000 Anxiety (CAM-S) Self-reported, Parent-reported, Research-reported: 0.000
Gil Piquer et al. [33] (2023)/Spain BLOOD DROW (Care centers) 1+	RCT n = 83 VRG = 43 (21 F, 22 M; 10, 7–12) CG = 40 (16 F, 24 M; 9, 7–12)	VRG: watch a movie titled “Henry” through an Oculus Quest 2 VR headset device (from the preparation to the moment of the puncture) CG: usual care	Pain: VAS Anxiety: GDS, NS Procedural time: minutes Post-intervention	Pain-VAS: 2.23 ± n.d. Anxiety-GDS Child: 1.58 ± n.d. NS-Nurse: 3.1 ± n.d. Procedural time: 1 (1–3)	Pain-VAS: 3.37 ± n.d. Anxiety-GDS Child: 2 ± n.d. NS-Nurse CG: 3.2 ± n.d. Procedural time minutes: 1 (1–2)	VRG vs. CG Pain (VAS): 0.012 Anxiety (GDS, NS): 0.081, 0.13 Procedural time (minutes): 0.77
Goktas and Avci [34] (2023)/Turkey DELTOID VACCINATION, VENIPUNCTURE, PHLEBOTOMY (Emergency department) 1++	RCT n = 144 VRG = 36 (18 F, 18 M; n.d.) KCG = 36 (18 F, 18 M; n.d.) MCG = 36 (18 F, 18 M; n.d.) CG = 36 (18 F, 18 M; n.d.)	VRG: VR distraction with the audio video “The Spacewalker”, using the dispositive Zore G07E VR Shinecon 3D Virtual Reality Glasses”, which includes a headset (starting 3 min before the procedure) KCG: a visual distraction technique with a kaleidoscope during the invasive procedure (starting 3 min before the procedure) MCG: a music distraction technique using twenty current popular songs played through the “S16 Bluetooth Over-Ear Wireless Headphones with Microphone” (starting 3 min before the procedure) CG: standard procedure	Pain: WBFPRS Anxiety: CAMS Fear: CMFS Post-intervention	Pain-WBFPRS: 0.42 ± 0.65 Anxiety-CAMS: 8.06 ± 1.90 Fear-CMFS: 14.78 ± 2.65	Pain-WBFPRS KCG: 0.67 ± 0.93 MCG: 0.67 ± 0.93 CG: 2.72 ± 1.32 Anxiety-CAMS KCG: 8.31 ± 1.74 MCG: 8.03 ± 2.57 CG: 7.47 ± 1.73 Fear-CMFS KCG: 14.81 ± 2.62 MCG: 14.14 ± 1.91 CG: 13.94 ± 2.16	VRG vs. KCG vs. MCG vs. CG Pain (WBFPRS): <0.001 Anxiety (CAMS): <0.001 Fear (CMFS): <0.001
Gold et al. [35] (2018)/USA BLOOD DRAW (Children’s hospital) 1+	RCT n = 143 (107 analyzed) VRG = 70 (33 F, 37 M; 15.79 ± 3) CG = 73 (38 F, 35 M; 15.06 ± 3.23)	VRG: VR game “Bear Blast” (applied VRä) using the Samsung Galaxy S6 mobile-based Gear VR goggles (aged 13–21) or the Google Pixel mobile-based Merge VR goggles (aged 10–12) CG: standard of care	Pain: VAS, CAS Anxiety: VAS, FAS Post-intervention	Pain VAS, CAS, FPS-R Patient-report 1.31 ± 1.59, 1.58 ± 2.02, 1.40 ± 0.73 Caregiver-report 1.06 ± 1.72, 1.19 ± 1.57, 1.54 ± 0.88 Anxiety VAS, FAS Patient-report 1.90 ± 2.22, 0.28 ± 0.22 Caregiver-report 1.52 ± 2.03, 0.33 ± 0.22	Pain VAS, CAS, FPS-R Patient-report 1.93 ± 2.22, 2.00 ± 2.10, 1.70 ± 1.13 Caregiver-report 2.26 ± 2.68, 2.29 ± 2.38, 2.02 ± 1.30 Anxiety VAS, FAS Patient-report 2.48 ± 2.07, 0.40 ± 0.24 Caregiver-report 2.48 ± 2.63, 0.38 ± 0.25	Pain (VAS, CAS, FPS-R) Patient-report VRG: 0.001, <0.001, <0.001 CG: 0.053, 0.095, <0.05 Caregiver-report VRG: <0.001, 0.001, 0.001 CG: <0.01, <0.01, 0.07 Anxiety (VAS, FAS) Patient-report VRG: <0.001, <0.001 CG: <0.01, <0.001 Caregiver-report VRG: <0.001, <0.001 CG: <0.01, <0.05
Goldman and Behboudi [17] (2021)/Canada IV CATHETERIZATION PROCEDURE (Emergency department) 1++	RCT n = 66 VRG = 35 (13 F, 22 M; 4.4 ± 0.9) CG = 31 (17 F, 14 M; 4.7 ± 0.75)	VRG: VR game (Roller Coaster app) played using an Asus Zenfone 2 ZE551ML mobile device, VR goggles, and a virtual reality headset CG: standard of care	Pain: FPS-R Anxiety: VSA Procedural time: minutes Post-intervention	Pain-FPS-R: 2 (0–4) Anxiety-VSA: 2.4 (0–8) Procedural Time minutes: 5 (3–10)	Pain-FPS-R: 4 (2–6) Anxiety-VSA: 2.4 (0–7) Procedural Time: 7 (3–13)	VRG vs. CG Pain (FPS-R): 0.004 Anxiety (VSA): 0.51 Procedural Time: 0.34
Hsu et al. [10] (2022)/ Taiwan INTRAVENOUS PLACEMENT (Pediatric ward) 1++	RCT n = 134 VRG = 69 (37 F, 32 M; 9.81 ± 1.70) CG = 65 (43 F, 22 M; 10.22 ± 1.70)	VRG: immersive VR with HTC Vive VR headset and head-mounted display device of the VR Cosmos helmet. Two types of interactive sessions in the VR environment were used: an instructional play session (pre-injection) and an emotional catharsis play session (post-injection) CG: before the procedure, an educational picture book was provided on intravenous placement	Pain: WBSPF Fear: CFS Post-intervention	Pain-WBSPF Child: 1.33 ± 1.60 Caregiver: 1.13 ± 1.51 Fear-CFS Child: 0.28 ± 0.54 Caregiver: 0.36 ± 0.64	Pain-WBSPF Child: 2.06 ± 2.00 Caregiver: 2.68 ± 1.74 Fear-CFS Child: 0.65 ± 0.94 Caregiver: 0.95 ± 0.96	VRG vs. CG Pain (WBSPF) Child: 0.028 Caregiver: <0.001 Fear (CFS) Child: 0.004 Caregiver: <0.001
Orhan et al. [36] (2023)/Turkey VENIPUNCTURE (Pediatric outpatient clinic) 1−	RCT n = 102 VRG = 52 (32 F, 20 M; 9.75 ± 1.56) CG = 50 (30 F, 20 M; 9.94 ± 1.39)	VRG: VR game “‘VR Feel the Nature” with 4 different options (sightseeing in a village, park, forest, and windmill) using a VR headset (Bobo VR Z4 mini) connected to a mobile phone CG: routine practice	Pain: FPS-R Anxiety: STAIC Post-intervention	Pain-FPS-R: 1.46 ± 1.49 Anxiety-STAIC: 31.48 ± 7.30	Pain-FPS-R: 4.44 ± 2.26 Anxiety-STAIC: 32.4 ± 69.22	VRG vs. CG Pain (FPS-R): 0.001 Anxiety (STAIC): 0.553
Osmanlliu et al. [37] (2020)/Canada VENIPUNCTURE (IV PLACEMENT) (Emergency department) 1+	RCT n = 62 VRG = 31 (20 F, 11 M; 11.1 ± 2.9) CG = 31 (18 F, 13 M; 12.3 ± 3.0)	VRG: VR distraction using a videogame Dreamland Oculus Rift^®®^ with the Oculus Rift ^®^ device, in a sitting position (starting 3 min before the procedure) + local standard of care CG: local standard of care	Pain: NRS Anxiety: CFS Post-intervention	Pain-VNRS: 3 (1,6;2,4) Anxiety-CFS: 1 (0, 2; 1, 1)	Pain-VNRS: 3 (1,5.5;2,5) Anxiety-CFS: 2 (0, 3; 1, 3)	VRG vs. CG Pain (VNRS): 0.75 Anxiety (CFS): n.d.
Thybo et al. [38] (2022)/Denmark VENOUS CANNULATION (University hospital) 1++	RCT n = 106 VRG = 52 (16 F, 36 M; 5.9 ± 1.4) CG = 54 (10 F, 44 M; 5.8 ± 1.4)	VRG: a three-dimensional VR interactive game named “Freddy the Frog (3D)”, using the Oculus Go VR goggles CG: dual-dimensional game with a tablet or smartphone	Pain: WBSPF Procedural time: minutes Post-intervention	Pain- WBSPF: 20 (0–40) Procedural time minutes: 2 (1–2.7)	Pain-WBSPF: 20 (0–55) Procedural time (minutes): 1.83 (1–2)	VRG vs. CG Pain (WBSPF): 0.19 Procedural time: 0.72
Top and Ayyildiz [39] (2021)/Turkey BLOOD DRAW (Blood draw unit) 1−	RCT n = 80 (77 analyzed) VRG = 37 (19 F, 18 M; 4.86 ± 0.99) CG = 40 (22 F, 18 M; 4.70 ± 0.09)	VRG: a video watching by virtual reality glasses 2 min before starting the procedure, and continued until the end of the blood draw CG: usual procedure in the blood test room	Pain: FPS-R Procedural time: minutes Post-intervention	Pain-FPS-R: 3.82 ± 1.20	Pain-FPS-R: 6.96 ± 2.08	VRG vs. CG Pain (FPS-R): < 0.001
Van den Berg et al. [40] (2023)/Netherlands VENIPUNCTURE, INTRAVENOUS CATHETER, INJECTION (Pediatric clinic) 1−	RCT n = 138 (114 analyzed) VRG = 60 (32 F, 28 M; n.d.) CG = 54 (30 F, 24 M; n.d.)	VRG: medical hypnosis (audio script) through VR, using the G2 VR headset from PICO (software provided by SyncVR Medical) CG: medical hypnosis induced by a trained healthcare provider (audio script)	Pain: WBSPF Observed reported pain: NRS Fear: CFS Post-intervention	Pain-WBSPF: 0.0 (0.0–3.5) Observed reported pain-NRS: 0.0 (0.0–1.0) Fear CFS 0.0 (0.0–0.75) Observed reported 0.0 (0.0–0.0)	Pain-WBSPF: 2.0 (0.0–4.0) Observed reported pain-NRS: 0.5 (0.0–1.0) Fear-CFS Child: 0.0 (0.0–1.0) Observer reported: 0.0 (0.0–1.0)	VRG vs. CG Pain (WBSPF): 0.289 Observed reported pain (NRS): 0.062 Fear (CFS) Child: 0.203 Observed reported: 0.231
Walther-Larsen et al. [1] (2019)/USA VENIPUNCTURE (Pediatric unit) 1+	RCT n = 64 (59 analyzed) GVR = 28 (1 F, 27 M; a 10.9 ± 2.8) GC = 31 (6 F, 25 M; a 10.1 ± 2.2)	VRG: VR game “Seagull Splash” using Samsung Galaxy S6 mobile-based Gear VR goggles and controller in the hang not assigned for the procedure GC: Standard care. Topical numbing cream, positioning, and distraction	Pain: VAS Procedural time: minutes Satisfaction: 0 to 10 scale Adverse event: number Post-intervention	Pain-VAS: 27 (0–33) Procedural time: 1.75 (1.0–3.6) Satisfaction: 81 (68–100)	Pain-VAS: 15 (5–30) Procedural time: 2.0 (1.0–2.5) Satisfaction: 89 (73–100)	VRG vs. CG Pain-VAS: 0.23 Procedural time: 0.58 Satisfaction: 0.82
Wong and Choi [41] (2023)/China VENIPUNCTURE (Pediatric unit) 1++	RCT n = 149 VRG = 75(40 F, 35 M; 7.21 ± 2.45) CG = 74 (46 F, 28 M; 7.21 ± 2.49)	VRG: immersive VR intervention with 2 age-appropriate VR scenarios (self-designed cartoon character DD who was going to undergo venipuncture), carried out with a disposable headset and a head-mounted display connected to a smartphone CG: standard care	Pain: FPS-R Anxiety: CSAS-C Length of procedure: minutes Post-intervention	Pain-FPS-R: 2.24 ± 2.81 Anxiety- CSAS-C: −0.10 ± 1.2 Length of procedure minutes: 4.43 ± 3.47	Pain-FPS-R: 4.99 ± 3.95 Anxiety-CSAS-C: 0.10 ± 1.02 Length of procedure minutes: 6.56 ± 7.39	VRG vs. CG Pain (FPS-R): <0.001 Anxiety (CSAS-C): 0.03 Procedural time: 0.03
Yildirim and Gerçeker [42] (2023)/Turkey IV INSERTION (Pediatric emergency department) 1++	RCT n = 150 VRG = 51 (24 F, 27 M; 6.4 ± 1.6) BCG = 50 (23 F, 27 M; 6.7 ± 1.7) CG = 49 (24 F, 25 M; 6.4 ± 1.6)	VRG: immersive VR, wearing a virtual headset and using Oculus Rift VR and a Samsung Galaxy S7 mobile phone BCG: cold Buzzy device attached to the arm and ending 5 min after IV insertion was completed CG: no distraction device. Distraction carried out by asking questions	Pain: WBSPF, CAS-C Anxiety: CAM-S Fear: CFS Post-intervention	Pain-WBSPF: 5.6 ± 1.9 CAS: 5.9 ± 1.5 Anxiety-CAM-S: 5.2 ± 1.4 Fear-CFS: 3.0 ± 0.7	Pain-WBSPF BCG: 5.9 ± 1.6 CG: 6.2 ± 1.0 CAS BCG: 5.8 ± 1.5 CG: 6.0 ± 1.1 Anxiety-CAM-S BCG: 5.1 ± 1.5 CG: 5.5 ± 1.2 Fear-CFS BCG: 2.9 ± 0.5 CG: 3.2 ± 0.5	VRG vs. BCG vs. CG Pain (WBSPF, CAS) 0.206, 0.897 Anxiety (CAM-S): 0.246 Fear (CFS): 0.033

BCG: Buzzy device cold vibration control group; CAM-S: children’s anxiety meter; CAS: color analog scale; CFS: children’s fear scale; CG: control group; CVCG: cold and vibration control group; CI: confidence interval; CMFS: child medical fear scale; CSAS-C: Chinese version of the state anxiety scale for children; F: female; FAS: facial affective scale; FLACC: face, legs, activity, cry, and consolability; FPS-R: faces pain scale-revised; GDS: Groninger distress scale; GMG: game mobile group; IQR: interquartile range; KG: Kaleidoscope control group; M: male; MCG: music control group; n.d.: no data; NRS: numeric rating scale; NS: numeric scale; SD: standard deviation; STAIC: Spielberger state-trait anxiety inventory for children; STAI-C: state-trait anxiety inventory for children; TVCG: television control group; USA: United States; VAS: visual analogic scale; VRG: virtual reality group; VRpG: passive virtual reality group; VRORG: virtual reality—Ocean Rift group; VRRG: virtual reality—Rollercoaster group; VSA: Venham situation anxiety; VAT: visual analog thermometer; SUDS: subjective units of distress; VNRS verbal numeric rating scale; VPT: Venham picture test; VR: virtual reality; WBSPF: Wong–Baker faces pain scale.

**Table 2:** Degree of recommendation for the use of virtual reality.

Certainty Assessment							№ of Patients		Effect		Certainty	Outcome
№ of Studies	Study Design	Risk of Bias	Inconsistency	Indirect Evidence	Imprecision	Other Considerations	Virtual Reality	Control	Relative (95% CI)	Absolute (95% CI)		
9	RCTs	Serious	It is not serious	Serious	It is not serious	Strong association	450	446	-	MD −1.83 (−2.93 to −0.72)	⨁⨁⨁◯ Moderate	WBFS. Self-reported
4	RCTs	Serious	It is not serious	Serious	It is not serious	-	199	197	-	MD −2.61 (−4.00 to −1.23)	⨁⨁◯◯ Low	WBFS. Parent-reported
3	RCTs	Serious	It is not serious	Serious	It is not serious	-	130	132	-	MD −2.71 (−2.82 to −2.6)	⨁⨁◯◯ Low	WBFS. Nurse reported
10	RCTs	Serious	It is not serious	Serious	It is not serious	Very strong association	583	579	-	SMD −0.71 (−1.13 to −0.28)	⨁⨁⨁⨁ High	NRS
4	RCTs	Serious	It is not serious	Serious	It is not serious	-	210	214	-	MD −2.92 (−5.45 to −0.38)	⨁⨁◯◯ Low	CAM. Self-reported
3	RCTs	Serious	It is not serious	Serious	It is not serious	-	142	141	-	MD −3.87 (−6.99 to −0.75)	⨁⨁◯◯ Low	CAM. Parent-reported
6	RCTs	Serious	It is not serious	Serious	It is not serious	Strong association	314	310	-	MD −1.27 (−1.99 to −0.56)	⨁⨁⨁◯ Moderate	CFS. Self-reported
4	RCTs	Serious	It is not serious	Serious	It is not serious	-	227	225	-	MD −1.33 (−2.08 to −0.58)	⨁⨁◯◯ Low	CFS. Parent-reported
2	RCTs	Serious	It is not serious	Serious	It is not serious	-	104	104	-	MD −1.13 (−2.24 to −0.03)	⨁⨁◯◯ Low	CFS. Nurse reported
8	RCTs	Serious	It is not serious	Serious	It is not serious	Strong association	378	372	-	MD −0.05 (−0.13 to 0.03)	⨁⨁⨁◯ Moderate	Procedural time
5	RCTs	Serious	It is not serious	Serious	It is not serious	Strong association	313	299	-	MD 0.05 (−0.76 to 0.65)	⨁⨁⨁◯ Moderate	Satisfaction providers
3	RCTs	Serious	It is not serious	Serious	It is not serious	-	259	248	-	MD 0.81 (0.26 to 1.37)	⨁⨁◯◯ Low	Satisfaction parents

The risk in the intervention group (and its 95% confidence interval) is based on the risk assumed in the comparison group and the relative effect of the intervention (and its 95% confidence interval). CAM: Children’s anxiety meter. CFS: Children’s fear scale. CI: Confidence interval; NRS: numerical rating scale. RR: Risk ratio. MD: Mean difference. SMD: Standard mean difference. RCT: Randomized clinical trial. WBFS: Wong–Baker faces scale. GRADE Working Group grades of evidence. High certainty: We are very confident that the true effect lies close to that of the effect estimate. Moderate certainty: We are moderately confident in the effect estimate—the true effect is likely to be close to the effect estimate, but there is a possibility that it is substantially different. Low certainty: Our confidence in the effect estimate is limited—the true effect may be substantially different from the effect estimate. Very low certainty: We have very little confidence in the effect estimate—the true effect is likely to be substantially different from the effect estimate. CI = confidence interval; RCTs = randomized controlled trial; RR = relative risk; ⨁⨁⨁◯ = level of recommendation.

## Data Availability

Not applicable.

## Public Involvement Statement

There was no public involvement in any aspect of this research.

## Guidelines and Standards Statement

This manuscript was drafted against the PRISMA (preferred reporting items for systematic reviews and meta-analyses for Protocols) for Systematic Reviews.
